# A novel epigenetic regulation of circFoxp1 on Foxp1 in colon cancer cells

**DOI:** 10.1038/s41419-020-03007-6

**Published:** 2020-09-19

**Authors:** Yanwei Luo, Fengxia Liu, Jinqi Ma, Yunfeng Fu, Rong Gui

**Affiliations:** Department of Blood Transfusion, the Third Xiangya Hospital of Central South University, 410013 Changsha, Hunan China

**Keywords:** Colorectal cancer, Targeted therapies

## Abstract

Foxp1 is a tumor suppressor in colon cancer. However, circFoxp1 derived from Foxp1 is an oncogene. In this study, we aim to investigate the role of circFoxp1 in colon cancer and the regulatory mechanism between circFoxp1 and Foxp1. 78 human colon tumor tissues and the matched paracancerous tissues were collected. Quantitative polymerase chain reaction, immunohistochemistry, quantitative methylation-specific PCR, chromatin immunoprecipitation assay, CCK-8 assay, and Tumor xenograft in nude mice were performed. The expression of circFoxp1 was increased and Foxp1 was reduced in colon cancer tissues, which were associated with a poor overall survival rate of the patients with colon cancer. CircFoxp1 recruited DNMT1 to the promoter of Foxp1, leading to promotor hypermethylation, thereby inhibiting Foxp1 transcription. Interfering circFoxp1 by siRNA in SW620 cells significantly inhibited cell viability, while knockdown Foxp1 expression partially restored SW620 cell viability. In addition, knockdown of circFoxp1 significantly sensitized colon cancer cells to Capecitabine in vitro and vivo through regulating Foxp1. We discovered a novel epigenetic pathway that circFoxp1 regulated Foxp1 in colon cancer cells. CircFoxp1 may regulate DNA methylation and demethylation to coordinate colon cancer cell proliferation and participate in chemotherapy drug responses. Therefore, circFoxp1 may be a potential therapeutic target for colon cancer.

## Introduction

The forkhead box protein 1 (Foxp1) belongs to the P subfamily of the FOX (Forkhead Box) family. These proteins functions in embryo development, cell cycle regulation, metabolism, and immune regulation. The abnormal expression of these genes is associated with tumorigenesis^1^. Foxp1 is a key transcriptional regulatory factor in the development of B lymphocytes^2^. Banham et al. found for the first time that Foxp1 gene can be detected in various normal and tumor tissues, including renal cell carcinoma, colon cancer. The expression abundance of Foxp1 in cancer tissues was significantly lower than that in matched normal tissues. Therefore, Foxp1 may be a tumor suppressor gene^3^ However, emerging evidence discovers that Foxp1 may have a dual function in the development of human tumors. On the one hand, overexpression of Foxp1 induced by chromosomal translocation causes diffuse large B-cell lymphoma (DLBCL), suggesting that Foxp1 has the role of oncogene^4^. On the other hand, Foxp1 is located on chromosome 3p14.1, which is a tumor suppressor region; and the high expression of Foxp1 in human primary breast cancer is associated with a good prognosis, suggesting that Foxp1 is a tumor suppressor in breast cancer^5^. In addition, in androgen-related prostate cancer, hypoxia, androgen stimulation, and androgen receptor blockers may jointly affect the expression of Foxp1^6^. Takayama K et al. found that Foxp1 was an androgen-responsive factor that negatively regulated the androgen receptor signaling pathway^7^.

One study found that low expression of Foxp1 in colorectal cancer tissue was associated with low survival rates. However, little is known about the molecular mechanism of Foxp1 loss in colon cancer. Studies have found that the exons 8 to 11 of Foxp1 form a circular RNA molecule, circFoxp1. circFoxp1 is an oncogene in gallbladder cancer and promotes tumor progression by enhancing the Warburg effect^8^. It is unclear the regulatory mechanism between circFoxp1 and Foxp1. In this study, we found that the expression of circFoxp1 and Foxp1 in colon cancer tissues was negatively correlated, and circFoxp1 hypermethylated the promoter of Foxp1 by recruiting DNMT1, thereby inhibiting the expression of Foxp1, and ultimately promoting the colon cancer progress.

## Materials and methods

### Human specimen collection

The study collected tumor tissues from 78 patients with colon cancer and the matched paracancerous tissues from September 2014 to September 2019. All patients with colon cancer were confirmed by pathological diagnosis. The clinical characteristics of colon cancer patients were obtained through electronic medical records, including gender, age, tumor size, tumor metastasis, tumor differentiation, tumor staging, and therapeutic intervention, recurrence, and survival time. This study was approved by the ethics committee of the Third Xiangya Hospital, Central South University. The study methodologies conformed to the standards set by the Declaration of Helsinki. The experiments were undertaken with the understanding and written consent of each subject.

### Quantitative polymerase chain reaction

Quantitative polymerase chain reaction (qPCR) was performed as previously described^9^. Total RNA was extracted from tumor tissues or cells using Trizol reagent (Invitrogen). Real-time quantitative PCR was performed on ABI 7500 Real-Time PCR System (SeqGen, Inc., Torrance, CA, USA). SYBR Premix EX Taq ™ II (TaKaRa) was used for the reaction. The primers were used as: circFoxp1, forward: CTCCTCTGCACCTTCCAAGA, reverse: ATCATAGCCACTGACACGGG; Foxp1, forward: AACGGCAAAGAGGGAGCC, reverse: GGCATGCATAATGCCACAGG; E2F1, forward: ACAAGGCCCGATCGATGTTT, reverse: CTGCAGAGACAAGGTGAGCA; retinoblastoma1 (Rb1), forward: AGAAACAAACCAAAATGGGAGGT, reverse: GGGTGTTCGAGGTGAACCAT; androgen receptor (AR), forward: AGGCGACAGAGGGAAAAAGG, reverse: CTCGCAGCCAAAGGGAGTTA; β-catenin, forward: CTGAGGAGCAGCTTCAGTCC, reverse: ATTGCACGTGTGGCAAGTTC; GAPDH: TTCCCGTTCTCAGCCTTGAC. GAPDH was used as an internal control.

### Immunohistochemistry

The Immunohistochemistry (IHC) experiments were performed as our previous reported^10^. Briefly, the paraffin-embedded tumor tissues were cut into 4 μm. The tissues were retrieved in citric acid buffer (PH = 7.0), and then were blocked with normal goat serum (Boster Biological Technology, Wuhan, China) for 60 min at room temperature. The tissues were incubated with primary antibody (anti-Foxp1, cat no. Ab16645, 1:200 dilution, Abcam; anti-Ki67, cat no. Ab92742, 1:500 dilution, Abcam) overnight at 4 °C. The tissues were then then with a horseradish peroxidase (HRP) -conjugated anti-rabbit secondary antibody for 2 h at 37 °C. Finally, the sections were counterstained with hematoxylin.

The expression of Foxp1 and Ki67 was semi-quantitated by immunoreactivity scoring. The intensity of Foxp1 staining was scored as 0 (negative), 1 (weak), 2 (moderate), and 3 (intense) by two pathologists who were blinded to the experiments. The immunoreactivity score was calculated as the percentage of positive cells multiplied by the intensity of staining.

### Cell culture

Human colon cancer cell lines (LOVO, HCT116, SW480, SW620) and human normal colon epithelial cells (HCoEpiC) were purchased from China Type Culture Collection (Wuhan). All cells were cultured in RPMI-1640 medium supplemented with 10% FBS. The culture conditions were 37 °C, 95% humidity, and 5% CO_2_ in a constant temperature incubator.

### Immunoblotting

Cell protein was extracted using RIPA lysis buffer. Protein concentration was determined using an Enhanced BCA Protein Assay Kit (Beyotime, Shanghai, China). In all, 20 μg protein samples were separated by electrophoresis in 10% SDSPAGE, and the proteins were transferred to polyvinylidene fluoride (PVDF) membrane. The transferred PVDF membrane was blocked with 5% skim milk for 2 h at room temperature, and then incubated with the primary antibody (anti-Foxp1, cat no. ab196978, 1:1000 dilution, Abcam; anti-GAPDH, cat no. ab181602, 1:3000 dilution, Abcam;) at 4 °C overnight. After washing three times with TBST, the membranes were incubated with secondary antibody goat anti-rabbit IgG antibody (1:4000) for 2 h. Finally, the signal of bands was developed with an ECL chemiluminescence kit (BeyoECL Plus, Beyotime, Shanghai, China).

### Viral constructions and infection

The plasmid expressing circFoxp1 was synthesized by Sangon (Shanghai, China). The target sequence of circFoxp1 small interfering RNA (siRNA) (siRNA sequence, 5′-TGACACGGGAACTTTAGAAATGATT-3′) was synthesized by Sangon (Shanghai, China). The plasmids were packaged into adenoviruses using the AAVPrime AAV System (GeneCopoeia, Inc.) according to the manufacturer’s protocol. The cells were infected with viral at multiplicity of infection = 50 for 48 h.

### Promotor activity measurement

Promotor activity was determined by luciferase reporter assay as our previous reported^11^. The full-length promoter of Foxp1 (2500 bp upstream to Foxp1 transcription start site), the truncated promotor fragments, and the mutant type fragments were synthesized by Sangon (Shanghai, China) and inserted into a pGL3-basic vector (Promega, Madison, WI, USA). The Dual Luciferase Reporter Gene Assay Kit (Beyotime, Shanghai, China) was used to assess luciferase activities following manufacturer’s protocol. SW620 cells were co-transfected with 50 ng pGL3-basic vector or pGL3-Foxp1, together with circFoxp1 expressed plasmid or siRNA. After transfection for 48 h, luciferase activity was determined on microplate reader.

### Quantitative methylation-specific PCR

Foxp1 promoter methylation status was measured by MSP. The cell genomic DNA was extracted using Genomic DNA Purification Kit (Thermo Scientific, CA). EZ DNA Methylation-Gold Kit (Zymo, Orange County, CA, USA) was used to modify the genomic DNA with bisulfite. The bisulfate-treated DNA was used to quantitative methylation-specific PCR (MSP). The following thermocycling conditions were used for the qPCR: initial denaturation at 95.0 °C for 3 min; 39 cycles of 95.0 °C for 10 s and 60 °C for 30 s. The primers were as following: methylated-specific primer, forward, 5′- CGGATTGATATGTTAGTTTTTAGGC -3′, reverse, 5′- AAATTTTCTCCCCTATTTCTCGA -3′; unmethylated-specific primer, forward, 5′- TGGATTGATATGTTAGTTTTTAGGTGT- 3′, reverse, 5′- AAAAATTTTCTCCCCTATTTCTCAA -3′.

### Chromatin immunoprecipitation assay

Chromatin immunoprecipitation (ChIP) assay was used to examine the binding of DNMTs to the Foxp1 promoter and was performed using the method as described previously^11^. In brief, circFoxp1-overexpressing and vector control cells were fixed with 1% formaldehyde and with glycine for 10 min to generate DNA–protein cross-links, and then sonicated on ice for 180 s to generate chromatin fragments. The chromatin fragments were immunoprecipitated with antibody (DNMT1, DNMT2, DNMT3a, DNMT3b, Abcam). The samples immunoprecipitated with IgG were used as control. An aliquot of cell lysates was served as the input DNA control. Precipitated DNA was subjected to quantitative PCR analysis.

### RNA pull-down

The biotin-coupled RNA complex was pulled down by incubating the cell lysates with streptavidin-coated magnetic beads (Invitrogen, Carlsbad, USA) following the manufacturer’s instructions. The bound proteins were eluted from the packed beads and the enrichment of DNMT1 was analyzed by western blotting. CircFoxp1 junction probe: 5′- AAAGGUUAGUAAAGAUUUCAAGGGCACAGUCACCGA-3′. Control probe: 5′- UUCAACAGCAGCAGCUUCAAGAGU-3′.

### CCK-8 assay

Cell survival rates were measured using Cell Counting Kit-8 (CCK-8) (Beyotime, Shanghai, China). Briefly, the cells were transfected with circFoxp1 siRNA or together with Foxp1 siRNA for 48 h. Then 0.5 × 104 cells were seeded in each 96-well plate for 24 h. In all, 10 μl CCK-8 reagents were added to each well at 1 h before the endpoint of incubation. OD 570 nm value in each well was determined by a microplate reader. To determine the effects of circFoxp1 on Capecitabine (CAPE)-induced growth inhibition, the transfected cells were treated with a series concentration (0, 1, 10, 20, 40, 80, 160 μg/L) of for 24 h and used to perform CCK-8 assay as above mentioned.

### Tumor xenograft in nude mice

Animal experiments were approved by the Ethical Committee for Animal Research of Central South University. The BALb/c nude mice (male, 2-month age, *n* = 5 mice/group) were purchased from Slac Laboratory Animal (Shanghai, China). The animal experiments were performed as our previous reported with some modifications^11^. The mice were randomly divided into three group: control group, scramble + CAPE group, and circFopx1 siRNA + CAPE group. The SW620 cells were transfected with circFopx1 siRNA or scramble control as above mention for 48 h prior to injections. To assess tumor growth and CAPE sensitization, 100 μl of the transfected SW620 cells (1 × 10^6^) was subcutaneously injected into nude mice. On day 14 following the injection of cells, the mice were intravenously injected with CAPE every 3 days with three times (3 mg/kg/injection). The control mice were injected with an equal volume of saline. Two lab assistants who blinded to the experiments measured tumor sizes regularly and calculated the tumor volume using the following formula: 0.5 × L × W^2^, where L and W refer to the length and width of the tumor, respectively. On day 26 following cell injections, the mice were sacrificed by injecting an overdose of pentobarbital sodium. The tumor tissues were removed and fixed with 4% paraformaldehyde and paraffin-embedded for the immunohistochemistry.

### Statistical analysis

All experiments were repeated at least three times, and data are expressed as the mean ± standard error of the mean (s.e.m.). Graphpad prism software (version 8, GraphPad Software, Inc., San Diego, CA, USA) was used to perform statistical analysis. Differences between two groups were compared by an independent-samples two-sided *t*-test. Differences among three or more groups were compared by one-way analysis of variance with a post hoc Bonferroni test. The Kaplan–Meier method was used to analyze overall survival and relapse time. *P* < 0.05 was considered to indicate a statistically significant difference.

## Results

### Expression of circFoxp1 and Foxp1 in colon cancer

**Fig. 1:**
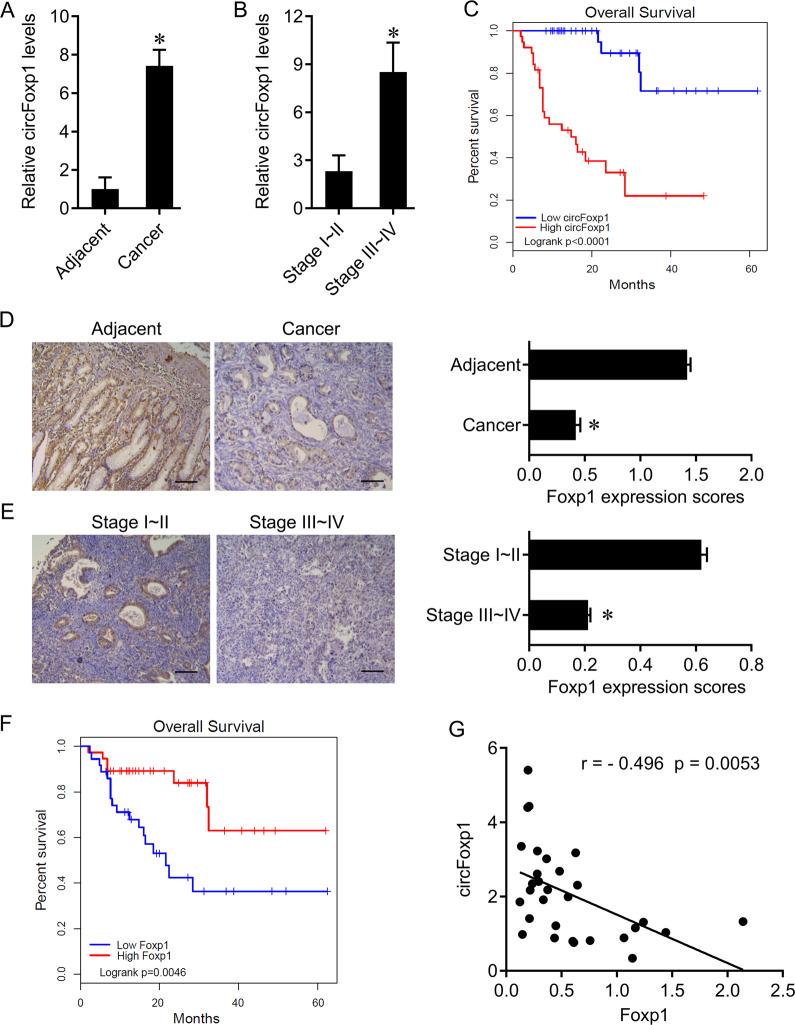
The expression of circFoxp1 and Foxp1 in colon cancer tissues. **a** circFoxp1 expression was quantitated by qPCR in colon cancer tissues and the matched adjacent tissues. **b** The expression of circFoxp1 in stage I-II and stage III–IV colon cancer tissues. **c** The association between circFoxp1 expression and survival rate in patients with colon cancer. **d** Foxp1 expression was semi-quantitated by immunohistochemical staining and was evaluated as the expression score. Scan bar = 200 μm. **e** The expression of Foxp1 in stage I–II and stage III–IV colon cancer tissues. Scan bar = 200 μm. **f** The association between Foxp1 expression and survival rate in patients with colon cancer. **g** Pearson correlation analysis was used to determine the correlation of circFoxp1 and Foxp1 in colon cancer. *p < 0.05 in colon cancer tissues as compared with their adjacent tissues.

In order to study the role of circFoxp1 and Foxp1 in colon cancer, we used qPCR and IHC to detect the expression of circFoxp1 and Foxp1 in colon cancer tissues. We found that circFoxp1 was significantly elevated in colon cancer tissues compared to paracancerous tissues (Fig. 1a), and the expression in stage III–IV was significantly higher than that in stage I–II (Fig. 1b). In addition, when analyzing the relationship between the expression of circFoxp1 and the overall survival of colon cancer patients, it was found that the higher the expression of circFoxp1, the lower the overall survival rate of the patients (Fig. 1c). In contrast, the expression of Foxp1 was significantly reduced in colon cancer tissues (Fig. 1d), and the expression in stage III–IV was significantly lower than that in stage I–II (Fig. 1e); and survival analysis also found that the higher Foxp1 expression, the higher the patient’s overall survival rate (Fig. 1f). Furthermore, correlation analysis found that there was a significant negative correlation between Foxp1 and circFoxp1 expression in colon cancer tissues (Pearson *r* = −0.496, *p* = 0.0053). These results suggest that Foxp1 and circFoxp1 play an important role in the development of colon cancer, and there may be some regulatory relationship between them.

### CircFoxp1 inhibits Foxp1 expression

**Fig. 2:**
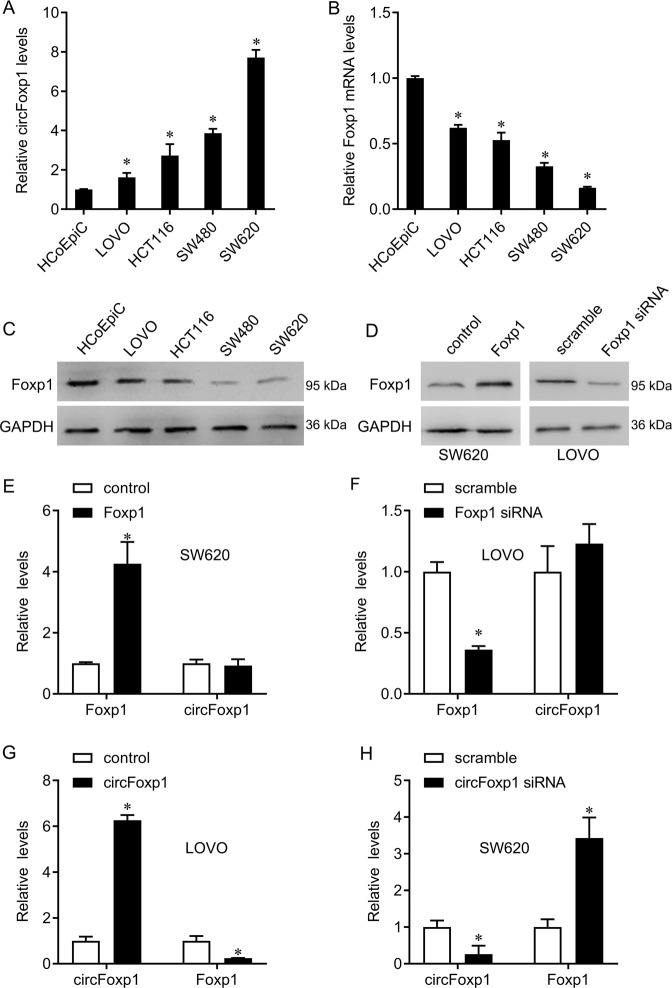
circFoxp1 suppresses Foxp1 level in colon cancer cells. **a** Expression of circFoxp1 in colon cancer cells. *p < 0.05 in colon cancer cells as compared with HCoEpic cells. **b** Expression of Foxp1 mRNA in colon cancer cells. *p < 0.05 in colon cancer cells as compared with HCoEpic cells. **c** Expression of Foxp1 protein in colon cancer cells. **d** Expression of Foxp1 protein in SW620 cells after Foxp1 transfection and in LOVO cells after Foxp1 siRNA transfection. **e** Expression of circFoxp1 and Foxp1 mRNA in SW620 cells after Foxp1 transfection. **f** Expression of circFoxp1 and Foxp1 mRNA in LOVO cells after Foxp1 siRNA transfection. **g** Expression of circFoxp1 and Foxp1 mRNA in LOVO cells after circFoxp1 transfection. **h** Expression of circFoxp1 and Foxp1 mRNA in SW620 cells after circFoxp1 siRNA transfection. *p < 0.05 compared with control.

We further study the regulatory relationship between circFoxp1 and Foxp1. Compared with normal colonic epithelial cells, the expression of circFoxp1 in colon cancer cells was significantly up-regulated, while the mRNA and protein expression of Foxp1 was significantly down-regulated (Fig. 2a–c), confirming their negative correlation. Since Foxp1 expression is highest in LOVO and lowest in SW620 in tumor cells, we overexpressed Foxp1 in SW620 cells and knocked down Foxp1 in LOVO cells (Fig. 2d) to observe the effect of Foxp1 on circFoxp1 expression. However, we found that Foxp1 overexpression or knockdown did not seem to significantly affect the expression of circFoxp1 (Fig. 2e, f). Interestingly, we knocked down circFoxp1 in SW620 cells and overexpressed circFoxp1 in LOVO cells, and found that circFoxp1 can negatively regulate Foxp1 expression (Fig. 2g, f).

### CircFoxp1 methylates Foxp1 promoter by recruiting DNMT1

**Fig. 3:**
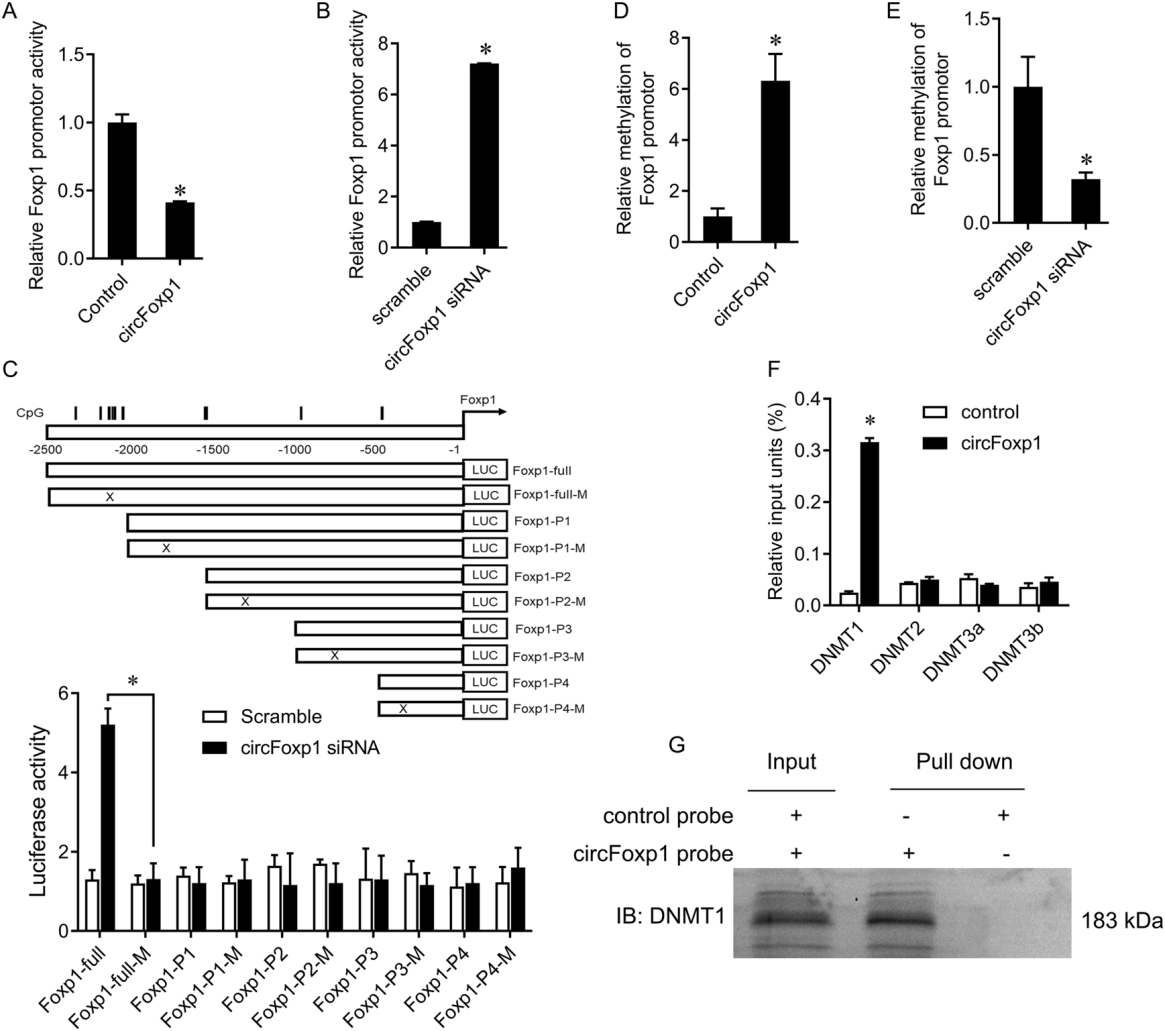
circFoxp1 methylated Foxp1 promotor. **a** Dual-luciferase report gene assay was performed to determine the promotor activity of Foxp1 in SW620 cells after circFoxp1 transfection. **b** Dual-luciferase report gene assay was performed to determine the promotor activity of Foxp1 in SW620 cells after circFoxp1 siRNA transfection. **c** Schematic diagrams of the CpG islands along the Foxp1 promoter. Numbers indicate the nucleotides relative to the TSS of Foxp1. Vertical lines indicate CpG dinucleotides. A series of truncated fragments and their mutants (indicated by “×”) fragments of Foxp1 promotor (Upper panels). Low Panels: Luciferase reporter activities of SW620 cells transiently co-transfected for 48 h with luciferase reporter constructs containing the wild-type sequence of Foxp1 promoter or its mutant counterparts, together with circFoxp1 or scramble control. **d** MSP was performed to determine the methylation status of Foxp1 promotor in SW620 cells after circFoxp1 transfection. **e** MSP was performed to determine the methylation status of Foxp1 promotor in SW620 cells after circFoxp1 siRNA transfection. **f** immunoblot analysis of Foxp1 after pulldown assay showing its specific association with circFoxp1. **g** ChIP was performed using the DNMTs antibodies or IgG control, qRT–PCR was performed to evaluate the specificity of protein binding in Foxp1 promotor. *p < 0.05.

We next investigated the mechanism by which circFoxp1 inhibits Foxp1 expression. Previous studies have shown that circRNA can regulate the expression of target genes through epigenetic modification. We here found that circFoxp1 can inhibit the expression of Foxp1 mRNA, suggesting that circFoxp1 may regulate the expression of Foxp1 at the transcriptional level. Therefore, we tested the activity of the Foxp1 promoter after overexpression or knockdown of circFoxp1. The results of the luciferase reporter gene showed that overexpression of circFoxp1 was significantly suppressed, while knockdown of circFoxp1 significantly increased the activity of the Foxp1 promoter (Fig. 3a, b). In addition, we further determined the specific effect of circFoxp1 on the Foxp1 promoter. By truncating and mutating the promoter fragment, we found that circFoxp1 mainly acts on the fragment region of the Foxp1 promoter −2500 to −2000, which enriched the CpG island, suggesting that the inhibitory effect of circFoxp1 on the Foxp1 promoter is related to methylation. Further MSP experiments showed that overexpression of circFoxp1 could significantly increase the methylation level of Foxp1 promoter, while knockdown of circFoxp1 significantly inhibited the methylation level of Foxp1 promoter (Fig. 3d, e). DNMTs play an important role in promoter methylation. Therefore, we used ChIP experiments to detect the enrichment of DNMTs in the Foxp1 promoter. The results showed that overexpression of circFoxp1 significantly increased the enrichment of DNMT1, but not DNMT2, DNMT3a, DNMT3b, on the Foxp1 promoter (Fig. 3f). From RNA pull-down assays, we observed that circFoxp1 was pull-downed with abundant DNMT1 protein (Fig. 3g), suggesting that circFoxp1/DNMT1 form an RNA-protein complex. These results indicate that circFoxp1 recruits DNMT1 to the Foxp1 promoter, leading to hypermethylation and inactivation.

### Silencing circFoxp1 inhibits colon cancer cell growth

**Fig. 4:**
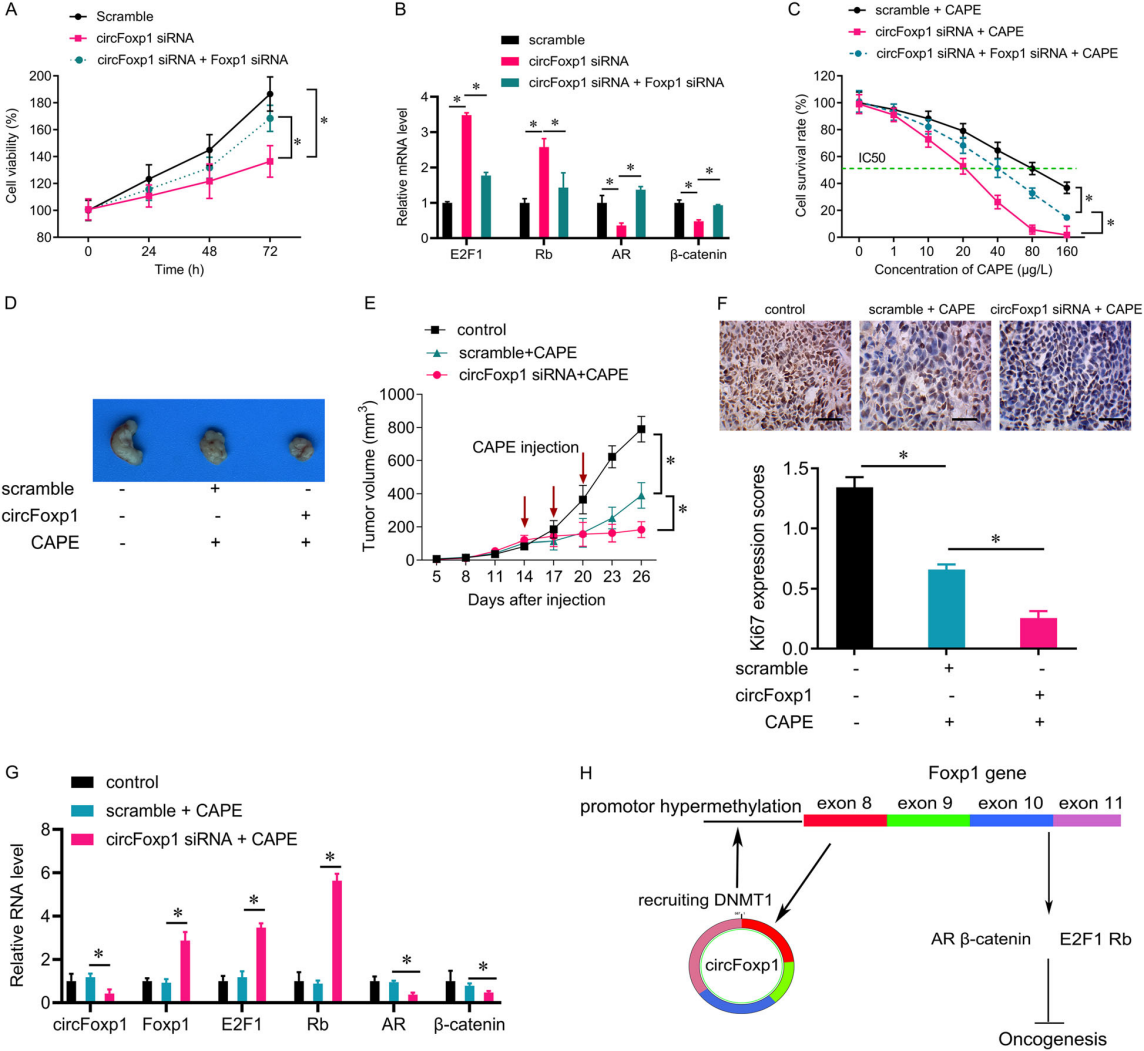
Silencing circFoxp1 inhibits proliferation of colon cancer cells. **a** CCK-8 was performed to measure the cell viability of SW620 cells after circFoxp1 siRNA transfection with or without Foxp1 siRNA. **b** mRNA expression of E2F1, Rb, AR, and β-catenin in SW620 cells after circFoxp1 siRNA transfection with or without Foxp1 siRNA. **c** SW620 cells were transfected with circFoxp1 siRNA alone, or together with Foxp1 siRNA, and then treated with Capecitabine (CAPE). CCK-8 was performed to measure the cell viability. **d** SW620 cells transfected with circFoxp1 siRNA and used for injection. On day 14 after cell injection, the mice were received CAPE injection every three days for three times. Mice were euthanized and tumors obtained from mice on day 26 after injection. **e** The tumor volumes were measured every 2-day. *p < 0.05. **f** The expression of Ki67 in tumor sections evaluated by IHC. Bar = 100 μm. *p < 0.05. **g** The expression levels of circFoxp1, Foxp1, E2F1, Rb, AR, and β-catenin in xenografted tumor tissues. *p < 0.05. **h** Putative model of circFoxp1 in colon cancer. Foxp1 produces circular RNA circFoxp1. Through the interaction with Foxp1 promoter, circFoxp1 recruits DNMT1 and induces extensive DNA methylation in the CpG islands. Working together, circFoxp1 inactivates Foxp1, which in turn promotes tumor cell proliferation in colon cancer.

We further investigated the biological function of circFoxp1 in colon cancer cells. We found that interfering circFoxp1 by siRNA in SW620 cells significantly inhibited cell viability, while knockdown Foxp1 expression partially restored SW620 cell viability (Fig. 4a), suggesting that circFoxp1 promotes colon cancer cell proliferation by inhibiting Foxp1. In addition, we detected the downstream target gene expression of Foxp1 by qPCR. Silencing circFoxp1 can significantly increase the expression of the tumor suppressor genes E2F1 and Rb, while suppressing the expression of the oncogenes AR and β-catenin, while simultaneously interfering Foxp1 significantly reduced the effect of silencing circFoxp1 (Fig. 4b). Furthermore, we investigated the role of circFoxp1 in capecitabine treatment, a first-line chemotherapy drug for colon cancer. The results showed that knockdown of circFoxp1 could significantly enhance the proliferation inhibition effect of CAPE on SW620 cells, while knockdown of Foxp1 could reduce the drug-sensitizing effect of silencing circFoxp1 (Fig. 4c). We also validated the biological role of circFoxp1 in vivo. The experiments in vivo showed that knockdown of circFoxp1 significantly promoted the sensitivity of colon cancer cells to CAPE, evaluated by a significant decrease in tumor volume (Fig. 4d, e) and a significant decrease in the expression of tumor growth marker Ki67 (Fig. 4f). We also confirmed that silencing circFoxp1 significantly upregulated the expression of Foxp1, E2F1, and Rb, while inhibited the expression of AR and β-catenin in vivo (Fig. 4g). These results suggest that circFoxp1 promotes oncogenesis by regulating its downstream target genes through Foxp1 (Fig. 4h).

## Discussion

This study demonstrates that Foxp1 acts as a tumor suppressor in colon cancer and a new mechanism for its regulation. We revealed that circular RNA circFoxp1 derived from Foxp1 exons promoted the proliferation of colon cancer cells. circFoxp1 bound to the Foxp1 promoter and recruits DNMT1 leading to hypermethylation, thereby inhibiting Foxp1 transcription. Although the specific mechanism is unknown, circFoxp1 induces extensive DNA methylation of the Foxp1 promoter, thereby suppressing its expression to promote proliferation. Studies have shown that silencing the expression of circFoxp1 can impair the differentiation of mesenchymal stem cells^12^; overexpression of circFoxp1 can promote the proliferation and metastasis of gallbladder cancer cell^8^. Foxp1 is a member of the Fox transcription family, which can transcriptionally regulate many target genes, including the tumor suppressor genes E2F1 and Rb, as well as the oncogenes AR and β-catenin^13–15^. In this study, we found that interfering Foxp1 by siRNA reduced the expression of E2F1 and Rb, and promoted the expression of AR and β-catenin, suggesting that Foxp1 is a tumor suppressor gene in colon cancer. This contrasts with the oncogenic role of Foxp1 in B-cell lymphoma. Therefore, Foxp1’s function is cell and tissue specific, and its specific mechanism remains to be explored. Overall, like other transcription factor members of the Foxp family, Foxp1 regulates the expression of oncogenes, tumor suppressor genes, and other genes, and is involved in cell proliferation and response to chemotherapy drugs.

In this study, we identified a new patterner of Foxp1, circFoxp1. CircFoxp1 is a 587 bp circular RNA consisting of Foxp1 exons 8–11 and is involved in mesenchymal stem cell differentiation and hepatocellular carcinoma cell proliferation and metastasis^8,12^, We here found that circFoxp1 recruited DNMT1 by binding to its host gene Foxp1 promoter. DNMT1 is a member of DNA methylation transferase and is important for maintaining DNA methylation. In mammals, the genomic methylation process is performed by DNA methyltransferases DNMT1, DNMT2, DNMT3a, and DNMT3b^16^. Among the three methyltransferases, DNMT3A and DNMT3B are called de novo methyltransferases and are responsible for establishing patterns and genomic imprint DNA methylation during embryogenesis. DNMT1 is essential for maintaining DNA methylation in the genome. During DNA replication, DNMT1 recognizes the hemi-methylated CpG as a substrate and restores the parental DNA on a specific methylated intact replicating strand^17^. By inducing promoter DNA hypermethylation, circFoxp1 inhibited Foxp1 transcription.

In this study, we found that circFoxp1 could inhibit the expression of Foxp1, but overexpression or knockdown of Foxp1 had no significant effect on the expression of circFoxp1. The mechanism that regulates circFoxp1 production is unclear. The current study finds that the formation of circular RNA is regulated by multiple factors, including heterogeneous ribonucleic acid proteins (hnRNPs) and SR proteins (proteins containing long repeating serine and arginine amino acid residues)^18^. There are also some proteins or molecules involved in the regulation of circular RNA. For example, ADAR (double-stranded RNA (dsRNA) -specific adenosine deaminase that prevents the activation of the innate immune system binds double-stranded RNA to adenine edited as hypoxanthine, and ATP-dependent RNA helicase A (also known as DHX9) inhibits the biogenesis of circular RNA by base pairing between inverted repeats^19^. In addition, epigenetic changes in histones and genomes can affect alternative splicing, but also may cause a direct impact on the biological occurrence of circular RNA^19^. For example, a recent study showed that knockdown of DNMT3B altered the circular RNA expression, but was not related to changes in the linear host gene expression^20^. A recent study found that the transcription factor SOX9 can significantly upregulate the expression of hepatocellular carcinoma circFoxp1^21^. Therefore, the production of circFoxp1 is regulated by a variety of factors, which may be cell-specific. So, it needs to be further studied to address the specific mechanism by which regulating circFoxp1 in colon cancer cells.

## Conclusion

We discovered a novel epigenetic pathway that circFoxp1 recruits DNMT1 to hyper-methylate the promoter Foxp1 in colon cancer cells. CircFoxp1may regulates DNA methylation and demethylation to coordinate colon cancer cell proliferation and participate in chemotherapy drug responses. Therefore, these data suggest that circFoxp1 may be a potential therapeutic target for colon cancer.
